# Cognitive and affective control for adolescents in care versus their peers: implications for mental health

**DOI:** 10.1186/s13034-023-00668-x

**Published:** 2023-11-09

**Authors:** Rosie McGuire, Sarah L. Halligan, Susanne Schweizer, Jovita T. Leung, Rachel M. Hiller

**Affiliations:** 1 Department of Psychology, University of Bath, Bath, United Kingdom; 2 Department of Psychiatry, University of Cape Town, Cape Town, South Africa; 3 School of Psychology, University of New South Wales, Sydney, Australia; 4 Department of Psychology, University of Cambridge, Cambridge, United Kingdom; 5 Institute of Cognitive Neuroscience, University College London, London, United Kingdom; 6 Division of Psychology and Language Sciences, University College London, 26 Bedford Way, London, WC1H 0AP United Kingdom; 7 Anna Freud National Centre for Children and Families, London, United Kingdom

**Keywords:** Care-experience, Affective control, Emotion regulation, Mental health, Post-traumatic stress

## Abstract

**Background:**

Many adolescents who have been removed from the care of their biological parent(s) and placed in State or Local Authority care have experienced significant adversity, including high rates of maltreatment and other trauma(s). As a group, these young people experience far higher rates of mental health difficulties compared to their peers. While their mental health outcomes are well-documented, little is known about mechanisms that may drive this. One potential mechanism, linked to both trauma and adversity exposure and mental health, is affective control (the application of cognitive control in affective contexts).

**Methods:**

We compared cognitive and affective control in 71 adolescents (65% girls) in care aged 11–18 (*M* = 14.82, *SD* = 2.10) and 71 age and gender-matched peers aged 11–19 years (*M* = 14.75, *SD* = 1.95). We measured cognitive and affective control using standard experimental tasks, and for those in care, we also examined associations with self-reported emotion regulation, mental health, and school well-being.

**Results:**

After controlling for IQ, there was a significant group difference in affective control performance, with those in care on average performing worse across all tasks. However, further analyses showed this was driven by deficits in overall cognitive control ability, and was not specific to, or worsened by, affective stimuli. Further, we found no evidence that either cognitive or affective control was associated with emotion regulation abilities or the mental health and well-being of young people in care.

**Conclusions:**

Results suggest that cognitive and affective control may not underlie mental health for young people in care, though limitations should be considered. We discuss implications for theory and intervention development, and avenues for further research.

*Trial registration*: https://doi.org/10.17605/OSF.IO/QJVDA

**Supplementary Information:**

The online version contains supplementary material available at 10.1186/s13034-023-00668-x.

Most young people who have been removed from the care of their biological parent(s) and placed in State care (referred to as Local Authority care in the UK and the Child Welfare System in the US) have experienced significant trauma and adversity. For many young people in care, this has often involved prolonged maltreatment (abuse and neglect), while almost all have experienced significant adversity, including exposure to parental drug and alcohol abuse, parental mental illness, poverty, and violence [14]. Once in care many face ongoing instability and challenges, including separation from siblings and frequent changes in caregivers/placements, as well as a heightened risk of exploitation and further trauma exposure [14, 63]. This accumulation of difficult experiences can lead to the development of psychological difficulties that affect a range of outcomes, including relationships and schooling [46]. Meta-analytic reviews show that up to half of young people in care meet criteria for a psychiatric disorder Engler et al. [16, 36], and complex comorbidities are the norm [4, 18]. Yet, there is little evidence about the mechanisms that drive these outcomes. Such evidence is important for informing how we can best understand the needs of these young people and develop more targeted interventions and support [43].

The research domain criteria (RDoC) highlights transdiagnostic mechanisms that may underlie mental health [31]. One mechanism included within this framework is cognitive control, which is defined as the capacity to flexibly engage and disengage with information to achieve current goal-demands [49]. There are three proposed facets of cognitive control: (i) updating and monitoring of working memory, (ii) inhibition of dominant responses, and (iii) shifting between tasks or mental sets [48]. A review of cognitive neuroimaging studies with clinical populations versus non-clinical controls implicates impaired cognitive control across a range of mental health diagnoses, supporting its inclusion in the RDoc framework [45]. Beyond mental health there is also emerging evidence that cognitive control may be important for general well-being. Perhaps particularly relevant to children and adolescents, this includes associations between cognitive control and school performance, general school well-being and engagement, and broader emotion regulation abilities [6, 9, 24, 25, 33].

A 2017 systematic review highlighted the limited literature on broader cognitive functioning in children who had experienced foster care [19]. In their review of children who had either experienced foster care, homelessness or poverty, only three studies had focused on children with experience of care, making concrete conclusions difficult. Although there was some consistent evidence of working memory deficits in the other groups of adversity-exposed young people [19]. A more recent study of adopted children aged 4–8 years old, found lower cognitive control was associated with increased behavioural difficulties, though not emotional difficulties [52]. In general, there remains limited evidence for whether there are differences in cognitive control or general cognitive functioning for children in care versus their peers. In relation to cognitive control specifically, it is also unclear whether any deficits might be enhanced within affective contexts (i.e., affective control), or whether any potential deficit is associated with mental health.

The application of cognitive control in affective contexts (i.e., affective control) may be particularly important for the development of mental health difficulties following significant trauma and adversity. Affective control is hypothesised to be a core cognitive building block underlying emotion regulation [59], as it requires controlling attention to and cognitively changing the meaning of emotional stimuli [51]. There is extensive evidence that emotion regulation is impaired across a range of mental health difficulties [1, 29]. There is also evidence that each specific facet of affective control (updating, inhibition, and set shifting) is associated with mental health in general population and clinical samples. For example, deficits in updating affective working memory (i.e., monitoring and replacing emotional information stored in short-term working memory) and inhibiting affective material is associated with affective disorders in adolescents (e.g., depression, anxiety, and post-traumatic stress disorder) [7, 17, 39, 50, 65, 67–69]. In addition, reduced ability to shift from strong maladaptive responses or trauma-related stimuli to more context-appropriate regulatory strategies are related to both internalising (e.g., affective, anxiety, and trauma-related disorders) and externalising (e.g., addiction, behavioural problems) difficulties [1, 5, 53].

The literature also suggests that maltreated young people have poorer affective control than their non-maltreated peers, and that this may contribute to their increased risk of psychopathology through emotional processing, particularly emotion regulation Jenness et al. [32, 56, 58]. However, there is emerging evidence to suggest that whilst youth exposed to adversity have poorer cognitive control than their peers, they perform just as well as their peers at affective control [70]. This uncertainty surrounding differences between trauma-exposed youths and their peers on neutral and affective versions of these cognitive tasks warrants further investigation, particularly in young people in care, where even less is known about their affective control ability and its link to mental health.

In this study, we used a quasi-experimental approach to address two key research questions. First, we aimed to investigate whether there were group differences in affective control in children in care versus their peers (who were not in care), and whether this was driven by general cognitive control or enhanced in affective contexts. Second, focusing solely on those in care, we explored whether their affective control abilities were associated with mental health and well-being, and whether these associations were mediated by emotion regulation. We focused on four mental health and well-being outcomes: (i) internalising difficulties, (ii) externalising difficulties, (iii) posttraumatic stress symptom severity, and (iv) school well-being. Beyond general internalising and externalising difficulties, we chose to measure PTSD symptoms as this is a trauma-specific mental health outcome with rates substantially elevated among young people in care [18]. We explored school well-being because of well-established evidence that young people in care are often at a significant disadvantage to their peers within the school context, with far higher rates of school disengagement, dissatisfaction, and poor attainment [3, 66]. Moreover, poorer school functioning is associated with negative life outcomes such as unemployment and contact with the criminal justice system [40]. IQ was controlled for in all analyses, along with age and gender where we were not looking at matched group differences, as these are key potential determinants of underlying cognitive control [8, 10, 11].

As a secondary goal, we also aimed to explore whether emotion regulation might mediate any association between the cognitive or affective control capabilities of young people in care and their mental health. As previously highlighted this was due to theoretical frameworks suggesting cognitive control is a building block of emotion regulation [59], which is in turn a building block for mental health [1, 29, 31]. Together, the overall goal of this work was to explore whether affective control may be a useful transdiagnostic target for adolescents in care. Such evidence is necessary for both theory and intervention development.

## Methods

The study protocol was pre-registered on the open science framework [42]. Ethical approval was granted from the University of Bath Psychology Research Ethics Committee, with further agreements from participating local authorities. Ethical approval for the sample of children from the general population was granted from University College London (UCL) Research Ethics Committee.

## Participants

### Adolescents in care sample

We recruited 71 young people in care, via local authorities. Eligibility criteria for this sample were: (i) aged 11–18 years old; (ii) on a full care order (which a council can apply for if it believes a child is suffering or at risk of suffering significant harm) if under 16 years old (any care order if 16 + as the young person can provide their own consent); (iii) adequate English and intellectual capacity to complete questionnaires and understand instructions; (iv) absence of psychosis or severe current suicidal ideations. Young people could be in any type of placement (i.e., living with a foster carer, kinship carer, or in a residential home), as long as they met our other eligibility criteria. There were 188 young people originally consented by local authority staff, of whom 75 (40%) agreed to participate. There were 71 participants with useable data (due to a technical error with 4 participants). Further technical difficulties meant that data was lost for specific affective cognitive control tasks for some participants, which resulted in additional participants being excluded from some analyses (see Tables 1 and 3 for *n* included in MANOVA analyses). Due to ethical requirements we were unable to compare differences between those who did and did not participate of the original 188 who were consented by the local authority.

**Table 1:** Descriptive statistics of raw affective control scores for young people in care and the comparison group

Variables	Care			Comparison		
	*n*	*M* (*SD*)	*M*_*adj*_ (*SE*)	*n*	*M* (*SD*)	*M*_*adj*_ (*SE*)
DS accuracy	52	3.83 (1.06)	4.01 (0.17)	51	4.86 (1.46)	4.68 (0.18)
ES happy incongruent RT	52	1155.31 (276.62)	1134.40 (31.02)	51	893.10 (153.57)	914.42 (32.05)
ES sad incongruent RT	52	1176.85 (267.53)	1158.89 (30.70)	51	885.64 (159.25)	901.23 (31.02)
ASS accuracy	52	0.65 (0.25)	0.67 (0.03)	51	0.80 (0.17)	0.78 (0.03)

*DS* digit span, *ES* emotional Stroop, *RT* reaction time, *ASS* affective set shifting

### Community sample

The comparison sample was 71 participants taken from a larger sample (*n* = 231) of secondary school students. For full details of this sample see protocol [60, 61]. Comparison sample participants were selected based on age and gender matching to participants from the care group. Selection was blind to the participant’s performance on any of the tasks or questionnaires. We randomised the ID numbers from the comparison group, then consecutively picked age and gender matches for each young person in care.

### Power

Power analysis was based on the primary research question and MANCOVA analysis i.e., differences between young people in care and their peers in affective control ability, controlling for IQ. The analysis was conducted in G*power Erdfelder et al., [72], with power set at 80%, the alpha level at 0.05 and using a medium effect size (f^2^ = 0.18), based on a recent study that also investigated affective control and mental health [60, 61]. When conducting the a priori sample size calculation, we allowed for six affective control variables to be included in the primary analysis, along with IQ, and expected these variables to be highly correlated. This resulted in a required sample of 142 (71 per group), which was the number recruited. However, due to issues with data collection resulting in significant missing data, our sample size was reduced, thus MANCOVA analyses were likely underpowered.

## Measures

Three experimental tasks were administered to assess cognitive control and affective control, across the three domains: updating, inhibition, and set shifting. A measure of fluid intelligence was included in the assessment as a potential co-variate and self-report questionnaires assessed emotional and functional well-being outcomes. Except where otherwise stated, the following measures were completed by participants in both samples.

### Affective cognitive control tasks

#### Updating

A backward digit span task was used to assess the updating of working memory in affective relative to neutral contexts [60, 61]. Participants were presented digits (300 ms) in serial order starting at two digits. Following the final digit, participants entered the digits they saw in reverse order into a keypad. Each span level was presented twice and continued to increase as long as participants recalled all digits in reverse order for at least one of the two trials per span level, otherwise the task ended. To manipulate valence, these digits were presented over negative or neutral background images. The images were randomly selected from the Geneva Affective Picture Database [12]. The recall phase of the task was self-paced but if no response was detected after two minutes the trial was scored as incorrect and the next trial was then presented (or not, as per the progression rule).

#### Inhibition

Inhibition of interference was assessed using a modified version of the Stroop task [54]. This task required participants to indicate whether an adjective they saw was happy or sad. The words were superimposed on the image of a face that was either congruent in emotion with the adjective (e.g., word: jolly, face: smiling), incongruent (e.g., word: gloomy, face: smiling) or neutral. The neutral condition contained scrambled faces, which used the same sad or happy faces with the pixels of the image mixed up so that it was not possible to determine the valence, but the perceptual properties remained the same. A red or green border appeared around the image for 200 ms after each trial to provide feedback, indicating an error or correct response, respectively. Trials were self-paced up to 4 sec. If no response was detected at that point, a red border appeared, and the next trial was presented.

In this modified version of the Stroop task, we only used two emotion categories (the original also included anger) to adapt the level of difficulty for younger participants. We also included only four adjectives per emotion category, compared to eight in the original task. For happy they are jolly, glad, joyful and cheerful. For sad they are upset, gloomy, miserable and hopeless. For each emotion there were four faces, two of which were from adult actors and the other two from child/adolescent faces and 50% of the face stimuli were female. The face stimuli were derived from several different databases to provide a diverse stimulus set in terms of demographics and emotional expressions. The databases included are the Chicago Face Database [38], the Radboud Faces Database [35], the London Face Research Set [13], the Emotional Faces Stimulus Set [47], and the NIMH Child Emotional Faces Picture Set [15]. There were 96 trials in total with each of the four actors being paired with each of the eight adjectives in each of the three conditions (congruent, incongruent, and neutral).

#### Set shifting

The capacity to shift flexibly between task demands was assessed using a set-shifting task [60, 61]. The task was a version of the Madrid Card Sorting Task with affective as well as neutral conditions [2]. Participants were dealt a card, which they assigned to one of four decks according to three possible sorting rules: card colour, number of items and shape (neutral) or emotional expression (affective). Participants were instructed that the sorting rule changes randomly and to adopt a different sorting rule whenever they are informed that they have made an error. The rule switch occurred after six to nine trials (on average after eight trials). Each rule was presented twice in the neutral version and twice in the affective version leading to 96 trials, which was self-paced. If no response was provided within 1 min, the trial was recorded as an error. The presentation order of the affective and neutral versions was counterbalanced across participants. Performance on the task was operationalised as random errors. These errors occur on any trial in the series after the initial two trials (needed to establish the correct sorting rule) and are most reliably associated with mental health outcomes in young adolescents on this version of the task [60, 61].

### Scoring

For each of these tasks we investigated the raw scores in the affective trials. Consistent with the wider literature, we also created proportional difference scores to isolate affective control from cognitive control. These subtraction-based proportional difference scores subtract performance on neutral trials from performance of affective trials and divide the difference score by the neutral performance. The index is therefore computed to reflect only the relative effect of the affective condition compared to the neutral condition, controlling for any age-related or care-experience related variance in general task performance. This is commonly used in developmental investigations of affect-cognition interaction (e.g., [34, 37]).

Upon peer-reviewer request, we also calculated residualised difference scores, which were created by running a regression model with raw cognitive control scores (task performance in neutral conditions) as the predictor variable and raw affective control scores (task performance in affective conditions) as the outcome variable. The unstandardized residual from this regression then becomes the score. As this was not part of our pre-registered analytic plan, we have reported them in the Additional file 1 but note any differences within the main text.

We calculated the original subtraction-based difference scores used in the main analysis in the following ways:

#### Updating task

To compute affective updating, we subtracted their maximum digit span in the neutral condition from the digit span reached in the affective condition and then divided this by the neutral digit span. A negative score indicates that the participant remembered fewer digits in the affective condition compared to the neutral condition, which suggests poorer affective updating.

#### Inhibition task

Affective inhibition capacity was computed as the difference in reaction time on incongruent/congruent trials from neutral trials, divided by neutral trial reaction time. The incongruent versus neutral comparison is considered a better reflection of affective control, so only these variables will be used in the main analysis. However, both the congruent and incongruent variables are included in an analysis in the Additional file 1, as per protocol (see Additional file 1: Table S1). For both congruent and incongruent variables, reaction time was computed for happy and sad trials separately. For both indices, a higher score reflects poorer affective inhibition, indicating slowed reaction times in the affective relative to the neutral trials.

#### Set-shifting task

Affective set-shifting was computed by subtracting the proportion of random error in the neutral condition from the proportion of random error in the affective condition divided by the number of random errors in the neutral condition. For ease of interpretation, the proportion of random errors was transformed into proportion correct, so that a higher score on this index indicates greater affective set-shifting capacity. Only performance on the colour and number trials were considered, as trial sets including the item type as sorting rule were more perceptually difficult in the affective condition (i.e., emotional facial expression) than the neutral condition (i.e., shapes).

### Potential covariates

#### Intelligence quotient (IQ)

An adapted (for online delivery) version of the 12-item Raven’s Advanced Progressive Matrices was used to assess fluid intelligence [55]. Participants were told that they should complete the task as quickly as possible. When calculating the total score on this measure, we removed one of the items retrospectively as the image uploaded to the website for this question was of poor quality, so the correct answer was slightly ambiguous. This meant scores could range from 0 to 11 correct responses, with higher scores indicating higher levels of IQ.

#### Age and gender

This was collected from social workers at the time of consent, with age confirmed with young people at the time of data collection, as time may have passed.

### Emotion regulation, mental health and well-being measures with adolescents in care

#### Emotion regulation

We measured emotion regulation using the Difficulties in Emotion Regulation Scale (DERS). This 36-item scale assesses six dimensions of emotion regulation: (i) *non-acceptance*, the tendency to experience secondary negative emotions in response to negative emotions; (ii) *goals*, difficulties engaging with goal-directed behaviours; (iii) *impulse*, the ability to control one’s behaviour when experiencing negative emotions; (iv) *awareness*, the positive tendency to attend to emotions; (v) *strategies,* the perception that emotions cannot be controlled; (vi) *clarity*; an individual’s ability to correctly identify their emotions [23]. Items are measured on a 5-point scale from *“almost never”* to *“almost always”.* All subscales are scored so that higher values reflect greater difficulty with emotion regulation (items reflecting greater ease of emotion regulation are reverse-coded). As per standard scoring procedures, we summed all six subscales to create a total emotion regulation score, which could range from 0 to 180, with higher scores reflecting poorer emotion regulation. The scale had good internal consistency in our sample of adolescents in care (Cronbach’s *α* = 0.95).

### Strengths and difficulties questionnaire

Internalising and externalising was measured via the 25-item self-report version of the SDQ [20]. Each item is rated on a 3-point scale,0 (*not true)*, 1 (*somewhat true*), 2 (*certainly true*). The internalising subscale comprises 10 items, covering emotional problems and peer problems, while the externalising subscale has 10 items covering conduct problems and hyperactivity. There is also a 5-item scale on prosocial skills (not used here). Scores on the internalising and externalising subscales can range from 0 to 20, with higher scores indicating greater difficulties. The measure has good psychometric properties in adolescents, including good internal consistency and test–retest reliability [21]. In our sample, internal consistency was acceptable (Externalising subscale Cronbach’s *α* = 0.76; Internalising subscale Cronbach’s *α* = 0.78).

### Child and adolescent trauma screen

The CATS is a 20-item self-report questionnaire that assesses DSM-5 PTSD symptoms (PTSS). Each item is measured on a 0 (*never*) to 3 (*almost always*) scale, therefore total scores can range from 0 to 60, with higher scores indicating increased PTSD symptoms. The self-report measure has excellent internal consistency and a good fit with the four symptom clusters in the DSM-5 indicated using confirmatory factor analysis [57]. In the current sample of young people in care, internal consistency was excellent (Cronbach’s *α* = 0.93). The CATS also has a trauma history checklist, which was not used in this project to minimise potential for distress and disengagement (as this is a group with very high rates of trauma and adversity exposure).

### The school satisfaction survey from the multidimensional students’ life satisfaction scale

To measure functional well-being, we used the 8-item School Satisfaction Survey [30], which asks young people about how they feel at school (e.g., “I learn a lot at school”). Each item is scored on a 0 (*never*) to 3 (*almost always*) scale, meaning scores can range from 0 to 24, with higher scores showing greater school satisfaction. The internal consistency for this measure in our sample of young people in care was excellent (Cronbach’s *α* = 0.93).

## Procedure

This study used a quasi-experimental design to explore the affective control ability of young people in care compared to an existing cohort of age and gender-matched young people. Details of procedures followed when collecting the comparison secondary school data are reported in the protocol [60, 61]. The task protocols for the new data collection mirrored those used with the secondary school sample. A key difference was that the comparison group data collection took place within schools, so data was collected in a group setting, whereas the in-care data collection took place in the young person’s home. In both situations, the researcher explained the tasks at the beginning, then allowed young people to proceed and was available for questions during the session. Research home visits were halted in March 2020 due to Covid-19 restrictions. Therefore most of the sample completed the tasks on their computer at home without support from a researcher. That said, even pre-pandemic, many of the adolescents requested to complete the tasks independently, rather than with researcher support (*n* = 21 of the 71 adolescents in care had home visits). Participants completing the tasks independently were provided with the researcher’s email address and contact number so that they had the opportunity to ask questions before they began or during the tasks, if needed.

Young people in care were recruited via two local authorities in England. Social care teams were provided with information about the study via information sheets and meetings. Letters were sent out to potentially eligible young people or their caregivers, to allow them to opt out of being contacted about the project. Local authorities provided informed consent for eligible young people to participate and young people provided their own informed assent (or consent if 16 + years old). Young people had the option to complete the tasks independently, or to arrange a time for the researcher to visit or call to go through the procedure with them. All study tasks and questionnaires were completed online for both samples. For each task, the participants were asked to first watch a brief video that provided instructions. After watching the video, they were also given the option to complete a demo or go straight ahead to the task. Once the tasks were completed, the young person completed questionnaires in Qualtrics. The full assessment battery took approximately 30 min. Participants each received a £10 voucher as a thank you for their time.

## Data analysis

To explore group differences in affective control, we ran two one-way MANOVAs, first with the raw scores of the affective control performance from the three tasks (see earlier scoring section), then with the proportional difference scores. The proportional difference score allows us to explore the effect of the affective condition controlling for performance on the neutral condition (i.e., general cognitive control)—that is, whether any group difference is purely driven by performance in affective control. As requested by a reviewer, all analyses with proportional difference scores (originally pre-registered and calculated as subtraction-based difference scores- see *scoring* section above) were re-run with residualised difference scores. This has been reported in the Additional file 1, with any discrepancies noted in the main text. We then repeated this analysis as a MANCOVA, with IQ as a covariate, to understand if any differences were driven by potential group IQ differences. Significant group differences were followed up with univariate tests to identify which specific aspects of affective control were different between the groups. To adjust for multiple comparisons, we used a Bonferroni adjustment (*p* < 0.013).

When testing assumptions for the MANCOVAs, we found that raw and proportional difference scores from the set shifting task were not normally distributed, however attempted transformations did not improve the distribution, so we continued with the raw data. As a sensitivity check, we re-ran these analyses with non-parametric versions of the tests due to this non-normal distribution. We found no difference in the pattern of results (see Additional file 1: Table S4).We also found outliers which were removed before conducting analyses: for raw scores, one multivariate care group outlier (Mahalanobis distance > 18.47) and one outlier in the emotional Stroop task from the care group, and two outliers on the digit span task from the comparison group (± 3 SD); for the proportional difference scores one outlier in the emotional Stroop task happy incongruent condition from the care group, and two outliers from the care group and one from the comparison group on the set-shifting task.

The second aim was to explore whether affective control was associated with mental health and well-being, and whether this was mediated by emotion regulation. This aim was only focused on the sample of children in care, as the comparison group have come from a larger community research project. Bivariate and point-biserial correlation analyses were used to show the basic associations between all variables. We then conducted hierarchical linear regressions, with the following outcomes: internalising difficulties, externalising difficulties, PTSS, and school well-being. In each analysis, age, gender, and IQ were controlled for in the first step, then affective control scores were included as predictor variables. Again, first we used raw scores from the emotional stimuli conditions to measure affective control, then we re-ran analyses using the proportional difference scores to isolate the affective from the neutral cognitive component of the tasks, to understand whether any associations were uniquely driven by affective control. Performance on the tasks were entered in a single step in the model. We ran four regressions with the raw scores, and four with the proportional difference scores, covering the earlier listed outcomes. All assumptions were met to conduct analyses, except three high leverage points (above 0.2), which were removed, resulting in *n* = 68. Where there were significant associations, we then planned to use mediation analysis using the PROCESS MACRO, to explore whether emotion regulation mediated any association between affective control and well-being outcomes.

## Results

### Descriptive statistics

The overall sample were 71 participants in the in-care group, aged 11–18 years (*M* = 14.82, *SD* = 2.10) and 71 participants in the comparison group, aged 11–19 years (*M* = 14.75, *SD* = 1.95). Groups were matched on age and gender, and in both groups, there were more girls (*n* = 46) than boys (*n* = 25). Gender was associated with general internalising difficulties (based on SDQ) and PTSS, with girls more likely to experience increased difficulties than boys. Age was associated with higher emotion dysregulation (i.e., poorer emotion regulation), and greater difficulties across all of the mental health and well-being measures (i.e., internalising, externalising, PTSS, and school well-being), as well as higher IQ scores. Age and gender were not significantly associated with performance on the affective control tasks. See Additional file 1: Table S2 for associations and Additional file 1: Table S3 for descriptive statistics.

### Group differences in affective control between adolescents in care and their peers

First, we ran a MANOVA without controlling for IQ and found that there was a statistically significant difference between young people in care and their peers in affective control performance (measured by performance on affective stimuli conditions), *F* (4, 99) = 18.85, *p* < 0.001, Wilks’ λ = 0.57, partial η^2^ = 0.43. Univariate one-way ANOVAs with a Bonferroni adjustment (*p* < 0.013), showed that the comparison group had better affective control than the care group across all tasks, as indicated by higher accuracy scores on the digit span and set shifting tasks, and lower reaction time scores on the emotional Stroop task (see Table 2). When this was re-run controlling for IQ, the significant multivariate (*F* (4, 97) = 12.26, *p* < 0.001, Wilks’ λ = 0.66, partial η^2^ = 0.34) and univariate group differences were retained, but with smaller effect sizes (see Table 2). The only exception, was that there was no longer a significant group difference in performance on the set shifting task (*p* = 0.016). See Table 1 for adjusted means.

**Table 2:** Results of follow-up one-way ANOVAs and ANCOVAs (controlling for IQ) investigating differences between young people in care and the comparison group in raw affective control scores

Variables	ANOVA			ANCOVA		
	*F*	*df*	η^2^	*F*	*df*	η^2^
DS accuracy	17.85***	1, 102	0.15	6.78*	1, 100	0.06
ES happy incongruent RT	35.99***	1, 102	0.26	22.05***	1, 100	0.18
ES sad incongruent RT	46.67***	1, 102	0.31	32.29***	1, 100	0.24
ASS accuracy	14.00***	1, 102	0.12	6.04*	1, 100	0.06

*DS* digit span, *ES* emotional Stroop, *RT* reaction time, *ASS* affective set shifting

^*^p < 0.05

^**^p < 0.01

^***^p < 0.001

Table 3 shows the means and standard deviations, plus the adjusted means and standard errors (adjusted for IQ) for the proportional difference affective control scores for the care and the comparison samples. MANOVA analysis found that there was no statistically significant difference between young people in care and their peers on the combined proportional difference affective control scores, *F* (4, 88) = 0.91, *p* = 0.46, Wilks’ λ = 0.96, partial η^2^ = 0.04. A MANCOVA adjusted for IQ also found no difference between young people in care and their peers on the combined proportional difference affective control scores, *F* (4, 86) = 1.44, *p* = 0.23, Wilks’ λ = 0.94, partial η^2^ = 0.06.

**Table 3:** Descriptive statistics of proportional difference affective control scores for young people in care and the comparison group

Variables	Care			Comparison		
	*n*	*M* (*SD*)	*M*_*adj*_ (*SE*)	*n*	*M* (*SD*)	*M*_*adj*_ (*SE*)
DS accuracy	49	−0.061 (0.30)	−0.069 (0.04)	43	−0.063 (0.29)	−0.054 (0.05)
ES happy incongruent RT	49	0.035 (0.15)	0.036 (0.02)	43	0.013 (0.17)	0.012 (0.03)
ES sad incongruent RT	49	0.059 (0.16)	0.063 (0.02)	43	−0.005 (0.13)	−0.010 (0.02)
ASS accuracy	49	0.081 (0.35)	0.088 (0.05)	43	0.042 (0.28)	0.034 (0.05)

*DS* digit span, *ES* emotional Stroop, *RT* reaction time, *ASS* affective set shifting

Taken together, these results show that when using the proportional difference scores, there were no significant performance differences for young people in or out of care. These proportional difference scores isolate affective control from core cognitive control ability, as they are calculated by subtracting performance on neutral trials from performance on affective trials and dividing the difference score by the neutral performance. Thus, this shows that the significant group differences across facets of affective control shown in the analysis of the raw affective control variables are in fact driven by deficits in cognitive control irrespective of context (i.e., neutral vs. affective). Additional file 1: Table S5 presents descriptive statistics of raw cognitive control scores.

At reviewer request, we re-ran these analyses using residualised difference scores. We found that using these scores, there is a significant difference between youth in care and their peers in affective control, but this was only driven by performance on the emotional Stroop task in incongruent sad trials. Therefore, differences between groups across other tasks and conditions remained non-significant.

### Associations between affective control and emotional and functional well-being in adolescents in care

The basic correlations between variables are presented in Additional file 1: Table S2 (note, there was little evidence of robust basic correlations between task performance and mental health or well-being outcomes). In the hierarchical linear regressions controlling for age, gender and IQ (see Table 4 for overall model summaries), accuracy in affective stimuli conditions on the set shifting task (but not digit span accuracy or reaction times in the emotional Stroop task) was a significant predictor of internalising and PTSD symptoms, as well as externalising symptoms. None of the measures of performance on affective control tasks were unique significant predictors of school well-being (Table 5). Finally, after controlling for covariates, none of the proportional difference scores (which isolate affective control from cognitive control) were significant predictors of internalising, externalising, or PTSD symptoms, or school well-being (Table 6).

**Table 4:** Overall model summaries from regressions investigating the effect of affective control on mental health and well-being outcomes

	*R*^*2*^	*F*	*df*	*R*^*2*^_*adj*_
Internalising symptoms				
Affective trial scores	0.23	1.89	7, 44	0.11
Proportional difference scores	0.16	1.17	7, 42	0.02
Externalising symptoms				
Affective trial scores	0.23	1.87	7, 44	0.11
Proportional difference scores	0.12	0.79	7, 42	−0.03
PTSD symptoms				
Affective trial scores	0.40	4.13**	7, 44	0.30
Proportional difference scores	0.38	3.73**	7, 42	0.14
School well-being				
Affective trial scores	0.32	2.89*	7, 44	0.21
Proportional difference scores	0.27	2.17	7, 42	0.14

^*^p < 0.05

^**^p < 0.01

^***^p < 0.001

**Table 5:** Regression summaries of raw affective control scores predicting mental health and well-being outcomes for young people in care, controlling for age, gender and IQ in the first step

	∆*F*	*df*	∆*R*^*2*^	B	β	*t*
Internalising symptoms	1.47	4, 44	0.10			
DS accuracy				−0.55	−0.15	−1.05
ES happy incongruent RT				0.00	0.09	0.45
ES sad incongruent RT				−0.00	−0.17	−0.81
ASS accuracy				4.60*	0.29	2.09
Externalising symptoms	2.50	4, 44	0.18			
DS accuracy				−0.96	−0.28	−2.02
ES happy incongruent RT				0.00	0.19	0.92
ES sad incongruent RT				−0.00	−0.23	−1.15
ASS accuracy				4.44*	0.31	2.21
PTSD symptoms	2.36	4, 44	0.13			
DS accuracy				−1.31	−0.09	−0.77
ES happy incongruent RT				−0.00	−0.05	−0.27
ES sad incongruent RT				−0.00	−0.05	−0.29
ASS accuracy				20.31**	0.35	2.83
School well-being	1.29	4, 44	0.08			
DS accuracy				1.58	0.24	1.82
ES happy incongruent RT				0.01	0.23	1.21
ES sad incongruent RT				−0.00	−0.09	−0.46
ASS accuracy				−0.08	−0.00	−0.02

*DS* digit span, *ES* emotional Stroop, *RT* reaction time, *ASS* affective set shifting

^*^p < 0.05

^**^p < 0.01

^***^p < 0.001

**Table 6:** Regression summaries of proportional difference affective control scores predicting mental health and well-being outcomes for young people in care, controlling for age, gender and IQ in the first step

	∆*F*	df	∆*R*^*2*^	B	β	*t*
Internalising symptoms	0.42	4, 42	0.03			
DS accuracy				−0.06	−0.01	−0.03
ES happy incongruent RT				−5.00	−0.18	−1.25
ES sad incongruent RT				0.03	0.00	0.01
ASS accuracy				−0.15	−0.02	−0.10
Externalising symptoms	0.48	4, 42	0.04			
DS accuracy				−0.01	−0.00	−0.01
ES happy incongruent RT				−3.52	−0.13	−0.89
ES sad incongruent RT				−1.41	−0.06	−0.40
ASS accuracy				1.45	0.15	0.95
PTSD symptoms	1.56	4, 42	0.09			
DS accuracy				2.84	0.06	0.46
ES happy incongruent RT				−16.13	−0.16	−1.29
ES sad incongruent RT				−15.86	−0.18	−1.43
ASS accuracy				5.72	0.16	1.18
School well-being	0.72	4, 42	0.05			
DS accuracy				0.97	0.04	0.31
ES happy incongruent RT				9.29	0.20	1.43
ES sad incongruent RT				−5.58	−0.13	−0.97
ASS accuracy				−1.45	−0.08	−0.58

*DS* digit span, *ES* emotional Stroop, *RT* reaction time, *ASS* affective set shifting

^*^p < 0.05

^**^p < 0.01

^***^p < 0.001

### Emotion regulation as a mediator of affective control and mental health

Correlation analyses showed no significant associations between measures of affective control and self-reported emotion regulation. As expected, poorer emotion regulation was associated with poorer mental health across all measures, and with poorer school well-being (Additional file 1: Table S2). As affective control, across all tasks, was not significantly associated with both emotion regulation and mental health, we did not explore mediation models.

## Discussion

This quasi-experimental study investigated whether young people in care have poorer affective control ability than their peers, and whether this may underlie the complex emotional and functional well-being difficulties that this group can commonly experience. While youth in care showed lower performance on affective control tasks compared to their peers, this was driven by a deficit in overall cognitive control, rather than a unique deficit in affective control. In contrast to predictions and despite the evidence of a group difference, cognitive and affective control were largely not associated with the emotion regulation, mental health, or well-being of young people in care, including internalising and externalising difficulties, PTSS, and school well-being.

The primary aim of this study was to understand whether there may be group differences in the affective control abilities of young people in care versus their peers, and whether this was driven by general cognitive control or specific to affective contexts. We found a difference in raw affective control scores between groups, but further analysis controlling for performance in neutral cognitive control tasks showed there was no additional deficit in affective conditions. This means the difference was driven by general cognitive control and there was no evidence of additional deficits under affective contexts. This adds to the limited literature that has explored cognitive functioning among adversity-exposed youth, including those with experience of care [19, 52], and further provides evidence that youth in care may experience general cognitive control deficits compared to their peers. This could be explained by the early life adversity that is experienced by those in care [14], as research has found that early trauma may have a negative impact on the development of cognitive control abilities (e.g., [41]).

When we re-ran these analyses (from reviewer feedback) using residualised difference scores (rather than the pre-registered subtraction-based scores) we found a significant difference between youth in care and their peers in affective control, but this was only driven by performance on the emotional Stroop task in incongruent sad trials (see Additional file 1). Whilst this aligns with literature suggesting that exposure to maltreatment causes negative emotional stimuli to be more salient [22, 44], there remained no differences between groups across other tasks (digit span and set-shifting) and conditions (incongruent happy trials in the emotional Stroop task). Thus, overall the pattern remained that there was little evidence of deficits in affective control (beyond deficits in cognitive control). However, we cannot draw conclusions specifically for the emotional Stroop task (which measures inhibition), because our results differed depending on the analytic method used. Findings further highlight the need to develop and validate these tasks and scoring methods. One potential account for the absence of an impact of affective contexts may be due to the stimuli’s limited affective salience. That is, the stimuli may not have generated a strong enough emotional response in participants to impact task performance. This should be further investigated.

The other key aim of our study was to explore whether cognitive or affective control might be associated with the mental health and well-being of young people in care. Here we found that, despite evidence of cognitive control deficits, cognitive and affective control abilities were largely not associated with the emotion regulation, mental health, or well-being (specifically school well-being) of young people in care. These findings are in contrast to literature suggesting that difficulty applying cognitive control in affective contexts is particularly important for the development of mental ill-health (e.g., [5, 17, 50] [68, 69]. Research has also suggested that affective control could be an important factor underlying the increased risk of psychopathology in maltreated youth Jenness et al. [32, 56, 58]. Accuracy on the affective stimuli conditions of the set shifting task was the only facet of affective control associated with mental health. However, contrary to expected results, this was associated with increased accuracy on this task, indicating that better affective control was associated with increased internalising, externalising and PTSD symptoms. That said, the effect size of these associations was small and basic correlations were inconsistent, suggesting this may be an artefact of the variable, which measures proportion of correct responses, lacking a normal distribution. Again, this effect may also be driven by cognitive control, as significant findings no longer held using proportional difference scores, which take performance on neutral tasks into account. As all other affective control measures were unrelated to these mental health and school well-being outcomes, it suggests that overall affective control may not be an important transdiagnostic process for young people in care, at least in relation to the outcomes focused on here (internalising, externalising, PTSS, and school well-being). However, it should be noted that these tasks produced low between-participant variability, which may explain why they are problematic when used in correlational analyses to predict individual differences [26]. Future research could attempt to overcome this with a larger sample using structural equation modelling with all measures of affective control loading onto one latent factor to see whether this would be associated with a latent factor of emotion regulation and/or mental health. This would be further strengthened using longitudinal methods to draw clearer conclusions around whether cognitive/affective control is causally linked to mental health.

As expected, emotion regulation was related to emotional and school well-being for this group, with poorer emotion regulation associated with increased mental health difficulties and lower school well-being [1, 29]. This is a well-established relationship, and we have further highlighted the importance of emotion regulation to emotional and functional well-being here, for young people in care. There has been some suggestion that affective control (i.e., cognitive control applied to affective contexts) is essentially the cognitive building blocks that underlies the process of emotion regulation [51]. Our study found no evidence that performance on established affective or cognitive control tasks were associated with self-reported emotion regulation, measured using the Difficulties in Emotion Regulation Scale (DERS). This suggests that for youth in care, who have often had extremely complex life experiences (and may have ongoing complexities and risks), that cognitive and affective control are not important drivers of emotion regulation skills.

However, there may be some measurement issues that should be considered as possibly affecting these findings. Firstly, each of the three proposed facets of affective control (updating, inhibition, and set-shifting) underlie different elements of emotion regulation, which may be masked when investigating associations with emotion regulation as a whole, as we did in this study. For example, updating of working memory predicts cognitive reappraisal ability, a specific emotion regulation strategy Hendricks & Buchanan, [27]. We did not measure specific emotion regulation strategies in this study, and did not intend to explore relationships between the facets of affective control and individual elements of self-reported emotion regulation. Another possible issue could be self-report bias, whereby mental health and well-being, as well as emotion regulation, were self-reported, thus required the meta-cognitive ability to self-reflect and report, which could lead to conflated correlations between these variables [64]. The affective control tasks do not require this meta-cognitive ability. A final measurement issue to consider is that research suggests young people in care typically under-report on their mental health (e.g., [28]). Future research could gather mental health data from a range of sources, including the young person and a trusted adult.

A final finding to note, is that age was positively correlated with difficulties in emotion regulation, which is surprising given much of the literature suggests that emotion regulation ability improves with age, particularly across early and middle childhood. However, during middle adolescence, around age 15, emotion regulation is at its lowest [71]. As our sample spanned 11–18 years old, this could be a possible explanation for our findings. In our sample, the worsening emotion regulation may also reflect the worsening mental health, as we would expect across adolescents, but particularly for youth in care. Another consideration around this sample is that caregiving experiences are thought to play an important role in shaping emotion regulation during adolescence [62]. Considering the unique caregiving these young people have received, this could also go towards explaining our findings in this group.

There are various strengths to this study. Primarily, we have focused on a group of young people with significant need but where there is very limited quantitative evidence of potentially malleable drivers of mental health and well-being. Nevertheless, results should be considered in light of several limitations. First, whilst we collected data from a large sample of 71 youth in care and 71 matched peers, the final sample included in the analyses was smaller due to technical difficulties experienced during data collection (e.g., issues connecting to the internet, which was most apparent for the ‘in-care’ group due to inequality in access to technology). Thus, the group difference findings should be interpreted in light of the analysis being slightly underpowered. Second, and related to ecological validity, many adolescents completed these tasks independently and it is difficult to accurately establish the potential impact of their engagement in the tasks. We have tried to rule out disengagement by looking for outliers in the data, but it is possible that some random data remains, which could impact the findings. Most of the ‘in care’ sample completed the tasks independently online, largely due to a change in procedure resulting from the Covid-19 pandemic, and this may have impacted engagement. The study was not powered to investigate whether performance was impacted by the presence of a researcher. Moreover, there was a great deal of variation in how much young people engaged with the researcher irrespective of whether tasks were completed with or without a researcher being physically present. At some home visits there was minimal interaction between the young person and researcher, and in some cases when tasks were completed without the researcher present, the young person would call the researcher for assistance. It is also important to note that young people were asked to watch a task demonstration video before completing the tasks, but it was not possible to record whether they did watch this or skip it. Finally, we only captured well-being related to school, and other aspects of the multi-faceted concept of well-being were not measured. The current study focused on school well-being as this is frequently a core area of functional impairment for youth in care [3, 66]. In addition, we did not measure trauma exposure and thus cannot draw conclusions on how trauma exposure may be related to cognitive or affective control (which was not the purpose of the paper). However, there is well-established evidence that children and teens in care have experienced extremely high rates of abuse, neglect, and other traumas and adversities compared to their peers (e.g., [14]), which supports the assumption that our in-care sample would have experienced far greater trauma and adversity than a general UK school sample.

## Conclusions

In sum, we found evidence of a deficit in cognitive control for young people in care compared to their peers, but no evidence that this deficit was enhanced in affective contexts. Contrary to the RDoC framework, which highlights transdiagnostic mechanisms underlying mental health, we found little evidence that cognitive and affective control were associated with emotion regulation, mental health or school well-being in youth in care. However, limitations of the measures used should be considered when interpreting these findings. Further research is urgently needed to better understand potential malleable mechanisms that may underlie the range of complex mental health difficulties so commonly experienced by young people in care, so that we may develop targeted interventions for this group.

### Supplementary Information

**Additional file 1: Table S1.** Results of follow-up one-way ANCOVAs (controlling for IQ) investigating differences between young people in care and the comparison group in raw affective control scores and proportional difference scores. **Table S2.** Pearson’s and point-biserial correlations for key variables for the young people in care sample (n = 71). **Table S3.** Descriptive statistics and independent samples t-tests to compare Emotion Regulation, Mental health and Well-being score for sample of young people in care (n = 71) and their matched peers (n = 71). **Table S4.** Results of Kruskal–Wallis H tests and Quade’s tests (controlling for IQ) investigating differences between young people in care and their peers in raw affective control scores and proportional difference scores. **Table S5.** Descriptive statistics of raw cognitive control scores for young people in care and the comparison group. **Table S6.** Regression summaries of residualised difference affective control scores predicting mental health and well-being outcomes for young people in care, controlling for age, gender and IQ in the first step.

## Data Availability

The datasets used and/or analysed during the current study are available from the corresponding author on reasonable request.
