# Supplementary material for: Cognitive and affective control for adolescents in care versus their peers: implications for mental health
Source: Child Adolesc Psychiatry Ment Health. 2023 Nov 9;17:128. doi: 10.1186/s13034-023-00668-x (PMC10636895; doi:10.1186/s13034-023-00668-x)
Supplement: Supplementary file 1 — Additional file 1: Table S1. Results of follow-up one-way ANCOVAs (controlling for IQ) investigating differences between young people in care and the comparison group in raw affective control scores and proportional difference scores. Table S2. Pearson’s and point-biserial correlations for key variables for the young people in care sample (n = 71). Table S3. Descriptive statistics and independent samples t-tests to compare Emotion Regulation, Mental health and Well-being score for sample of young people in care (n = 71) and their matched peers (n = 71). Table S4. Results of Kruskal–Wallis H tests and Quade’s tests (controlling for IQ) investigating differences between young people in care and their peers in raw affective control scores and proportional difference scores. Table S5. Descriptive statistics of raw cognitive control scores for young people in care and the comparison group. Table S6. Regression summaries of residualised difference affective control scores predicting mental health and well-being outcomes for young people in care, controlling for age, gender and IQ in the first step. [file 13034_2023_668_MOESM1_ESM.docx]

**Additional material**

**Group differences in affective control between adolescents in care and their peers**

In the main MANCOVA analyses we included the incongruent variables from the emotional Stroop task, as this is considered a better reflection of affective control. However, we have also re-run the MANCOVA analyses here to include both the congruent and incongruent variables, as per protocol (removed for blinding). This MANCOVA analysis (adjusting for IQ) using the raw affective control scores found significant multivariate (*F* (6, 99) = 9.07, *p*< .001, Wilks’ λ= .65, partial η^2^= .36) and univariate group differences (see Additional Table 1). Again, the only exception, was that there was no longer a significant group difference (due to Bonferroni correction) in performance on the set shifting task (*p* = .018). The MANCOVA analysis using the proportional difference affective control scores found no statistically significant difference between young people in care and their peers, *F* (6, 88) = 1.35, *p*= .24, Wilks’ λ= .92, partial η^2^= .08. This replicates findings from our main analysis suggesting there is no difference when the congruent variables from the emotional Stroop are also included in the analysis.

Additional Table 1

*Results of follow-up one-way ANCOVAs (controlling for IQ) investigating differences between young people in care and the comparison group in raw affective control scores and proportional difference scores.*

| Variables | Raw scores | | | Proportional difference scores | | |
| --- | --- | --- | --- | --- | --- | --- |
|  | *F* | *df* | η^2^ | *F* | *df* | η^2^ |
| DS accuracy | 9.05** | 1, 104 | .08 | 0.02 | 1, 93 | .00 |
| ES happy incongruent RT | 24.49*** | 1, 104 | .19 | 0.92 | 1, 93 | .01 |
| ES sad incongruent RT | 31.86*** | 1,104 | .23 | 5.45* | 1, 93 | .06 |
| ES happy congruent RT | 25.59*** | 1, 104 | .20 | 0.99 | 1, 93 | .01 |
| ES sad congruent RT | 24.16*** | 1, 104 | .19 | 1.57 | 1, 93 | .02 |
| ASS accuracy | 5.76* | 1, 104 | .05 | 0.90 | 1, 93 | .01 |

*Note.* DS= digit span, ES= emotional Stroop, RT= reaction time, ASS= affective set shifting. *p < .05. **p < .01. ***p < .001

Additional Table 2

*Pearson’s and point-biserial correlations for key variables for the young people in care sample (n= 71*)*.*

| Variables | 1. | 2. | 3. | 4. | 5. | 6. | 7. | 8. | 9. | 10. | 11. | 12. | 13. | 14. | 15. |
| --- | --- | --- | --- | --- | --- | --- | --- | --- | --- | --- | --- | --- | --- | --- | --- |
| *Demographic characteristics* |  |  |  |  |  |  |  |  |  |  |  |  |  |  |  |
| 1. Age | - |  |  |  |  |  |  |  |  |  |  |  |  |  |  |
| 1. Gender | -.03 | - |  |  |  |  |  |  |  |  |  |  |  |  |  |
| 1. IQ | .34** | -.13 | - |  |  |  |  |  |  |  |  |  |  |  |  |
| *Raw AC scores* |  |  |  |  |  |  |  |  |  |  |  |  |  |  |  |
| 1. DS accuracy | .02 | .03 | .31* | - |  |  |  |  |  |  |  |  |  |  |  |
| 1. ES happy incongruent RT | -.12 | .18 | -.25* | .02 | - |  |  |  |  |  |  |  |  |  |  |
| 1. ES sad incongruent RT | -.13 | .07 | -.26* | .07 | .71*** | - |  |  |  |  |  |  |  |  |  |
| 1. ASS accuracy | -.04 | -.01 | .16 | .03 | .12 | .12 | - |  |  |  |  |  |  |  |  |
| *Prop difference AC scores* |  |  |  |  |  |  |  |  |  |  |  |  |  |  |  |
| 1. DS accuracy | -.12 | .06 | -.11 | .35** | -.00 | -.04 | .01 | - |  |  |  |  |  |  |  |
| 1. ES happy incongruent RT | -.03 | .01 | -.15 | .19 | .52*** | .16 | -.10 | .03 | - |  |  |  |  |  |  |
| 1. ES sad incongruent RT | .01 | -.04 | -.03 | .01 | -.01 | .34** | -.31* | -.12 | .07 | - |  |  |  |  |  |
| 1. ASS accuracy | .14 | .02 | .27* | -.07 | .03 | -.07 | .23 | -.08 | .13 | .03 | - |  |  |  |  |
| *Mental health outcomes* |  |  |  |  |  |  |  |  |  |  |  |  |  |  |  |
| 1. Internalising symptoms | .30* | -.24* | .12 | -.04 | -.05 | -.03 | .21 | -.06 | -.12 | .01 | -.08 | - |  |  |  |
| 1. Externalising symptoms | .08 | .16 | -.09 | -.10 | .14 | .14 | .29* | .05 | -.02 | -.09 | .05 | .38** | - |  |  |
| 1. PTSD symptoms | .35** | -.28* | .08 | -.06 | -.03 | .02 | .20 | .05 | -.05 | -.18 | -.04 | .54*** | .24* | - |  |
| 1. School well-being | -.49*** | -.01 | .02 | .13 | .05 | -.00 | -.07 | .10 | .02 | -.12 | -.16 | -.49*** | -.36** | -.29* | - |
| *Emotion regulation* |  |  |  |  |  |  |  |  |  |  |  |  |  |  |  |
| 1. DERS total | .26* | -.20 | -.02 | -.02 | .11 | .09 | .18 | .09 | .08 | -.09 | .04 | .68*** | .52*** | .71*** | -.48*** |

*Note.* AC= affective control, DS= digit span, ES= emotional Stroop, RT= reaction time, ASS= affective set shifting, DERS= difficulty in emotion regulation scale, ERQ-CA= emotion regulation questionnaire for children and adolescents. *p < .05. **p < .01. ***p < .001

Additional Table 3

*Descriptive statistics and independent samples t-tests to compare Emotion Regulation, Mental health and Well-being score for sample of young people in care (n= 71) and their matched peers (n=71).*

|  | *M* | *SD* | Range | *t(df)* |
| --- | --- | --- | --- | --- |
| Emotion regulation (DERS) |  |  |  | -14.24*** (101.27) |
| *Youth in care* | 97.83 | 28.52 | 46-170 |  |
| *Matched peers* | 44.27 | 13.84 | 17-88 |  |
| Internalising symptoms (SDQ) |  |  |  | -.003 (113.97) |
| *Youth in care* | 7.54 | 4.13 | 0-19 |  |
| *Matched peers* | 7.53 | 2.44 | 3-13 |  |
| Externalising symptoms (SDQ) |  |  |  | -2.54* (118.41) |
| *Youth in care* | 8.64 | 3.80 | 0-18 |  |
| *Matched peers* | 7.29 | 2.40 | 3-14 |  |
| PTSD symptoms (CATS) |  |  |  |  |
| *Youth in care* | 22.61 | 15.77 | 0-58 |  |
| *Matched peers* | - | - | - |  |
| School Well-being (SSS) |  |  |  |  |
| *Youth in care* | 20.49 | 6.99 | 8-32 |  |
| *Matched peers* | - | - | - |  |

DERS= Difficulties in Emotion Regulation Scale, SDQ= Strengths and Difficulties Questionnaire, CATS= Child and Adolescent Trauma Screen, SSS= School Satisfaction Survey. CATS and SSS not collected in control group. *p < .05. **p < .01. ***p < .001

**Group differences in affective control between adolescents in care and their peers (non-parametric tests)**

We re-ran these analyses investigating group differences with non-parametric tests to assure that findings did not change based on the methods of analysis, as some of the affective control data was not normally distributed. We found that all results were replicated (see Additional table 4 below).

Additional Table 4

*Results of Kruskal-Wallis H tests and Quade’s tests (controlling for IQ) investigating differences between young people in care and their peers in raw affective control scores and proportional difference scores.*

| Variables | Kruskal-Wallis H | | | Quade | | |
| --- | --- | --- | --- | --- | --- | --- |
|  | *F* | *df* |  | *F* | *df* |  |
| *Raw affective control scores* |  |  |  |  |  |  |
| DS accuracy | 25.79*** | 1 |  | 8.66** | 1 |  |
| ES happy incongruent RT | 36.47*** | 1 |  | 29.95*** | 1 |  |
| ES sad incongruent RT | 35.55*** | 1 |  | 30.04*** | 1 |  |
| ASS accuracy | 8.08** | 1 |  | 3.35 | 1 |  |
| *Proportional difference scores* |  |  |  |  |  |  |
| DS accuracy | .47 | 1 |  | .46 | 1 |  |
| ES happy incongruent RT | .16 | 1 |  | .12 | 1 |  |
| ES sad incongruent RT | .67 | 1 |  | 1.61 | 1 |  |
| ASS accuracy | .59 | 1 |  | 1.28 | 1 |  |

*Note.* DS= digit span, ES= emotional Stroop, RT= reaction time, ASS= affective set shifting. *p < .05. **p < .01. ***p < .001

Table 5

*Descriptive statistics of raw cognitive control scores for young people in care and the comparison group.*

| Variables | Care | |  | Comparison | |  |
| --- | --- | --- | --- | --- | --- | --- |
|  | *n* | *M* (*SD*) | *M_adj_* (*SE*) | *n* | *M* (*SD*) | *M_adj_* (*SE*) |
| DS accuracy | 57 | 4.23 (1.77) | 4.47 (0.22) | 57 | 5.37 (1.55) | 5.12 (0.22) |
| ES happy neutral RT | 57 | 1140.84 (288.41) | 1117.94 (32.94) | 57 | 871.42 (182.02) | 894.32 (32.94) |
| ES sad neutral RT | 57 | 1153.72 (296.81) | 1132.53 (32.48) | 57 | 887.12 (156.28) | 908.33 (32.48) |
| ASS accuracy | 57 | 0.64 (0.26) | 0.66 (0.03) | 57 | 0.79 (0.18) | 0.77 (0.03) |

*Note.* DS= digit span, ES= emotional Stroop, RT= reaction time, ASS= affective set shifting.

**Re-running analyses with residualised-difference scores rather than subtraction-based difference scores**

MANOVA analysis found that there was a statistically significant difference between young people in care and their peers on the combined residualised difference affective control scores, *F* (4, 94) = 4.11, *p* < .01, Wilks’ λ= .85, partial η^2^= .15. Univariate one-way ANOVAs with a Bonferroni adjustment (*p* < .013), showed that the comparison group had better affective control than the care group in the emotional Stroop task only, with significantly faster responses to incongruent sad trials (but not happy trials). A MANCOVA adjusted for IQ also found a difference between young people in care and their peers on the combined residualised difference affective control scores, *F* (4, 92) = 3.23, *p* < .05, Wilks’ λ= .87, partial η^2^= .12. The univariate group difference in the emotional Stroop task was also retained, with a significant difference between groups for the sad incongruent trials only, albeit with smaller effect sizes.

When investigating associations with mental health outcomes, after controlling for covariates, none of the residualised difference scores (which isolate affective control from cognitive control) were significant predictors of internalising, externalising, or PTSD symptoms, or school well-being (Additional Table 6).

Table 6

*Regression summaries of residualised difference affective control scores predicting mental health and well-being outcomes for young people in care, controlling for age, gender and IQ in the first step.*

|  | ∆*F* | | df | ∆*R^2^* | B | β | *t* |
| --- | --- | --- | --- | --- | --- | --- | --- |
| *Internalising symptoms* | 0.52 | | 4, 46 | .04 |  |  |  |
| DS accuracy | |  |  |  | -0.09 | -.02 | -0.16 |
| ES happy incongruent RT | |  |  |  | -0.00 | -.09 | -0.68 |
| ES sad incongruent RT | |  |  |  | 0.00 | -.01 | -0.09 |
| ASS accuracy | |  |  |  | 3.87 | .17 | 1.20 |
| *Externalising symptoms* | 1.43 | | 4, 46 | .11 |  |  |  |
| DS accuracy | |  |  |  | -0.21 | 0.06 | 0.41 |
| ES happy incongruent RT | |  |  |  | -0.00 | -0.12 | -0.84 |
| ES sad incongruent RT | |  |  |  | 0.00 | 0.02 | 0.13 |
| ASS accuracy | |  |  |  | 6.99 | 0.32 | 2.26* |
| *PTSD symptoms* | 1.77 | | 4, 46 | .10 |  |  |  |
| DS accuracy | |  |  |  | 0.23 | .02 | 0.13 |
| ES happy incongruent RT | |  |  |  | -0.01 | -.11 | -0.91 |
| ES sad incongruent RT | |  |  |  | -0.01 | -.13 | -1.04 |
| ASS accuracy | |  |  |  | 21.39 | .25 | 2.06* |
| *School well-being* | 0.79 | | 4, 46 | .05 |  |  |  |
| DS accuracy | |  |  |  | 0.85 | .12 | 0.94 |
| ES happy incongruent RT | |  |  |  | 0.00 | .04 | 0.28 |
| ES sad incongruent RT | |  |  |  | -0.00 | -.10 | -0.74 |
| ASS accuracy | |  |  |  | -6.25 | -.15 | -1.16 |

*Note.* DS= digit span, ES= emotional Stroop, RT= reaction time, ASS= affective set shifting. *p < .05; **p < .01; ***p <.001
